# Psychometric properties and concurrent validity of the Transgender Congruence Scale (TCS) in the Swedish setting

**DOI:** 10.1038/s41598-020-73663-3

**Published:** 2020-10-29

**Authors:** Stavros I. Iliadis, Cathrine Axfors, Agnes Friberg, Hans Arinell, Ulrika Beckman, Attila Fazekas, Louise Frisen, Lotta Sandström, Nils Thelin, Jeanette Wahlberg, Maria Södersten, Fotios C. Papadopoulos

**Affiliations:** 1 Department of Women’s and Children’s Health, Uppsala University, 751 85 Uppsala, Sweden; 2 Department of Neuroscience, Psychiatry, Uppsala University, 751 85 Uppsala, Sweden; 3 Department of Gender Dysphoria, Södra Älvsborgs Hospital, 441 30 Alingsås, Sweden; 4 Department of Psychiatry, Lund University, 285 21 Lund, Sweden; 5 Department of Clinical Neuroscience, Karolinska Institutet, 171 77 Stockholm, Sweden; 6 Department of Clinical Sciences, Umeå University, 901 87 Umeå, Sweden; 7 Division of Psychiatry, Linköping University Hospital, 581 85 Linköping, Sweden; 8 Department of Endocrinology and Dept. of Health, Medicine and Caring Sciences, Linköping University, 581 83 Linköping, Sweden; 9 Department of Clinical Science, Intervention and Technology, Karolinska Institutet, 141 52, Huddinge, Sweden

**Keywords:** Psychiatric disorders, Psychology

## Abstract

The Transgender Congruence Scale (TCS) is a non-binary tool used in Sweden for gender dysphoria (GD) assessment; however, its Swedish version has not been validated. To investigate the psychometric properties of the TCS, its capacity to distinguish individuals with GD and its concurrent validity compared to other scales. Patients with GD (n = 135) and controls (n = 443) filled in a questionnaire containing sociodemographic questions, the TCS, the Utrecht Gender Dysphoria Scale (UGDS), and the Gender Identity/Gender Dysphoria Questionnaire for Adolescents and Adults (GIDYQ-AA). TCS had good discriminatory validity and internal consistency. Patients with GD, stratified by birth-assigned sex, had lower TCS scores compared to controls. Confirmatory factor analysis (CFA) supported the two-factor model of the TCS*.* Multiple-group CFA suggested measurement invariance between birth-assigned sexes and configural invariance between patients with GD and controls. Area under the ROC curve for birth-assigned males was 0.991 and for females 0.994. A TCS mean value of three provided sensitivity 94.3% and 95.1% as well as specificity 98.6% and 98% for aM and aF, respectively. The TCS was significantly correlated to UGDS and GIDYQ-AA. The TCS may be a valuable tool in the clinical assessment of individuals with GD.

## Introduction

An individual’s sex at birth is determined based on genital appearance and is usually referred to as *birth-assigned sex* (*male; aM or female; aF*). Gender role behavior is closely related to a person’s gender identity, denoting the extent of experience of conformity to others of the same gender or the sense of being a male or female^1^. Gender Dysphoria (GD), as defined in the Diagnostic and Statistical Manual of Mental Disorders, 5th edition (DSM-5), refers to the conflict between a person’s assigned gender and the gender with which they identify^2^. The condition reflects the discomfort with the expected roles or the physical appearance that the assigned gender implies^3^.

The use of psychometric instruments in transgender persons who seek healthcare for gender affirming treatment is often included in the evaluation process. The purpose of the use is partly as a diagnostic aid and partly as a baseline measurement for follow-up in the future.

The Utrecht Gender Dysphoria Scale (UGDS) was introduced around 1997 and GD is treated as being caused by the divergence between sense of self-gender identity and body aspects^4,5^. The scale has been reported to be able to distinguish between transsexual and non-transsexual persons as well as those suitable for gender affirming surgery^4^.

The Gender Identity/Gender Dysphoria Questionnaire for Adolescents and Adults (GIDYQ-AA) is a binary scale developed by Deogracias et al.^6^ with a different approach. Authors aimed to capture somatic, sociolegal, social, and subjective dysphoria indicators. A person’s gender identity was treated as a bipolar continuum, with unproblematic gender identity and gender dysphoria being on the edges of the gender identity.

The Transgender Congruence Scale (TCS) was developed by Kozee et al. in an attempt to capture the transgender’s congruence with their own gender identity perception^7^. The scale was designed to approach gender identity through the scope of the transgender model, which does not pathologize transgender individuals’ experiences and perceptions, as opposed to previous scales, that utilized the transsexual model. In the transgender model, gender is more fluid and changeable, rather than binary, and thus more inclusive^7^. Moreover, the TCS can be utilized for the evaluation of patients that do not undergo both hormonal and surgical gender affirming treatment while the UGDS and GIDYQ scales lack this option because they comprise questions on specific sex characteristics. For these reasons, the non-binary character of the TCS scale can be applied to individuals before and after different modalities of gender affirming treatment as well as to persons with non-binary gender identity. Validation of the TCS scale could promote the use of this non-binary, short and easy to administrate scale in the context of clinical evaluation and prediction of outcomes in transgender people.

The aim of this study was to evaluate the psychometric properties of TCS and its capacity to distinguish individuals with gender dysphoria in the Swedish setting. A secondary aim was to examine the scale’s concurrent validity by comparing TCS with two more widespread and better-validated scales, the UGDS and the GIDYQ-AA.

## Methods

### Sample and procedure

The present project is part of the Swedish Gender Dysphoria Study (Svenska Könsdysforistudien; SKDS), an ongoing longitudinal multicenter study on gender dysphoria. SKDS aims to investigate how anamnestic, socio-demographic, biological, neurocognitive, and personality-related factors affect physical and mental health, social integration and quality of life in people with GD.

The SKDS study was initiated in 2016. All individuals with an ongoing health-care contact within psychiatry, endocrinology or gynecology for gender dysphoria evaluation or treatment at one of the participating centers in Alingsås/Gothenburg, Linköping, Lund/Malmö, Stockholm, Umeå and Uppsala are being asked about participation in SKDS. Exclusion criteria are age < 15 and inability to adequately communicate in Swedish. All potential participants receive oral and written information as well as a prepaid envelope and a form for obtaining consent. The recruitment rate of the SKDS study is 77%.

Study controls were recruited via email-based open invitations to medical students in Uppsala University as well as students in psychology, the Bachelor’s programme in personnel, work and organization and the psychology programs 1, 2 and 3 at Stockholm University. Additional recruitment was utilized with posters at university facilities, the public library and on social media (Facebook).

Questions on gender dysphoria topics that were not relevant for controls were excluded from the surveys or replaced with the question “have you been treated for gender affirming purposes now or in the past?”. Respondents that were diagnosed with GD or had undergone gender affirming treatment as well as those with answers that implied gender dysphoria were excluded from the control group.

Psychometric instruments (TCS, UGDS—aM or aF version, GIDYQ-AA—aM or aF version) were distributed during the second week after inclusion in both groups. The sex-specific UGDS and GIDYQ-AA version was determined from the answer on the birth-assigned sex question. In the case group, all participants were administered the TCS scale and either the UGDS or the GIDYQ-AA scale. In the control group, all participants received all three rating scales. For the TCS and UGDS scales, up to two missing answers were accepted in order to be included in the analyses, while the corresponding number for the GIDYQ-AA was five questions.

Until April 2019, 328 patients were recruited in the SKDS study. Of those, 90 were aM, 142 were aF and data on birth-assigned sex was missing for 96 persons who were therefore excluded from the present study. Furthermore, 80 persons (32 aM, 48 aF) had started hormonal treatment and were excluded due to the possible effect of the treatment on GD scales evaluation. Five individuals (2 aM, 3 aF) with missing data on this question were also excluded from the study sample. Finally, we excluded from further analyses individuals where no TCS data was available (2 aM and 7 aF) as well as those that had missing answers in > 2 statements in TCS (1 aF for the whole scale and 1 aF for the factor statements). Finally, one aF had nine missing answers in GIDYQ-AA and was thus excluded. Therefore, the group of patients with GD consisted of 135 study participants (53 aM, 82 aF).

In the control group, 561 individuals responded to the invitations and no data on birth-assigned sex was available for two persons who were thus excluded. Of the remaining 166 aM and 393 aF, 13 persons reported a legal sex different from their birth-assigned sex and were therefore also excluded. Furthermore, we excluded 11 individuals (3 aM, 8 aF) that reported previous gender affirming treatment, and one more person with missing relevant data. An additional subgroup of 29 persons was excluded due to self-reported diagnosis of GD (1 aF), unspecified gender identity disorder (3 aM, 1 aF) or ongoing GD investigation (7 aM, 17 aM). Furthermore, 12 individuals that expressed a wish for legal sex reassignment were not included in the final study sample. Finally, one aF with multiple missing answers on TCS as well as 47 persons (9 aM, 38 aF) lacking data on the whole TCS scale were also excluded. After excluding one aF with missing answers on seven items in UGDS, 443 study participants comprised the control group.

Ethical approval regarding patients with GD was obtained from the Regional Ethical Review Board in Uppsala (ethical approval for the SKDS study; reg. no. 2016-013 and 2016-013/1). Data on controls were anonymous; therefore, no ethical approval was necessary for this group. All research was performed in accordance with relevant guidelines and regulations. Informed consent was obtained from all participants.

### Measures

The UGDS scale is binary; hence existing in two difference versions, one for aM and the other for aF persons. It consists of 12 items with answers on a five-point Likert-type scale (points 1–5 per item) and focuses on gender identity and roles as well as body aspects. A higher point sum indicates GD and a threshold of 50 to 60 points has been suggested as indicative of severe GD^8^. To date, there are no published studies on the performance of UGDS in a Swedish setting, but preliminary unpublished data from our group suggests good psychometric properties when validated against the Gender Identity/Gender Dysphoria Questionnaire for Adolescents and Adults (GIDYQ-AA) (Finndin et al. 2015, unpublished data).

The GIDYQ-AA scale consists of 27 questions with five-point Likert-type possible answers (1–5 points; always, often, sometimes, rarely, never as possible answers), for the previous 12 months, and a lower calculated mean value points towards GD. Two different versions for aM and aF exist. As reported by the authors, a threshold of three points would have a 90.4% sensitivity and 99.7% specificity to identify GD ^6^. The scale has been validated by Singh et al. with excellent sensitivity and specificity figures^9^. The performance of GIDYQ-AA in a Swedish setting has been only validated by unpublished work (Christopoulou et al. 2014, unpublished data).

The TCS scale consists of 12 statements about the past two weeks with five Likert-type possible answers (1–5 points per statement). The scale measures two factors: *appearance congruence* (statements 1–9), examining whether external appearance adequately represents gender identity, and *gender identity acceptance* (statements 10–12), which reflects the extent of perceived gender identity acceptance as opposed to the assigned by the society gender identity. A low mean score is associated with higher symptoms of GD. To meet the need for a validated instrument that can evaluate treatment results in persons who have undergone different kinds of gender affirming surgery in Sweden, the TCS scale was translated following the World Health Organization’s recommendations^10^, after permission from the authors. Thereafter, the scale was tested in 26 persons in various phases of evaluation and treatment where the majority of participants perceived the scale as non-binary and easy to complete^11^.

Participants were asked about their education level (dichotomized as primary school/high school vs. university/college) and occupation (employed, sick leave, unemployed, parental leave, other). Satisfaction with body appearance was measured on a 100-point Likert-type scale (“how satisfied are you with your body?”; 1 = very unsatisfied, 100 = very satisfied), as did gender incongruence (“to what extend is your body’s appearance in line with your inner identity?”; 1 = completely in line, 100 = not at all). Furthermore, participants were asked about their sexual orientation (attracted only to men, attracted mostly to men, attracted equally to men and women, attracted mostly to women, attracted only to women, asexual, uncertain, other) and categorized as androphilic (attracted only/mostly to men), gynephilic (attracted only/mostly to women) or bisexual (attracted to men and women equally).

### Statistics

Univariate differences in sociodemographic data between patients with GD and controls were examined by use of chi-square test for binary variables. The satisfaction with body appearance and gender incongruence scores were not normally distributed (Kolmogorov–Smirnov and Shapiro–Wilk tests). Thus, the non-parametric Mann–Whitney *U*-test was applied.

For each respondent, a TCS and GIDYQ-AA mean score was calculated, where lower mean value suggests stronger gender dysphoria. Regarding UGDS, scores were instead summed up, higher sum being indicative of gender dysphoria. The questions with a reverse score were inverted before calculating response points.

To calculate the internal consistency of the TCS scale, McDonald’s ω was used^12^. A confirmatory factor analysis (CFA) was performed to test the construct validity of the TCS instrument for the total study sample. Due to ordinal data structure, the DWLS (Diagonally Weighted Least Squares) estimator was used. Comparative Fit Index (CFI), Standardized Root Mean Square Residual (SRMR) and Root Mean Square Error of Approximation (RMSEA) were used as model fit indices. CFI ≥ 0.95, SRMR < 0.08 and RMSEA < 0.06 were considered as an indication of good fit^13^.

To evaluate the factor structure of the TCS scale across subgroups, two multiple-group confirmatory factor analyses (MGCFA) with mean structures in order were executed. Birth-assigned sex as well as patients with GD and controls were used as subgroups. For each MGCFA, we started with no constraints on factor loadings or item intercepts, which served as the baseline model. Next, we ran the MGCFA with factor loadings constrained to equality. In a third step we constrained the item intercepts to equality. Due to inequality in sample sizes, ΔCFI < 0.01 and ΔRMSEA < 0.015 were used when comparing changes in model fit indices^14^.

Comparison of TCS scores (for the total scale and separately for the *appearance congruence* and *gender identity acceptance* factors and for each TCS question), as well as UGDS and GIDYQ-AA scores, between patients with GD and controls, was performed separately for aM and aF with Mann–Whitney *U*-test, since the scales’ scores did not follow a normal distribution, neither in the total study sample nor within patients with GD and controls (Kolmogorov–Smirnov and Shapiro–Wilk tests). Moreover, differences in TCS scores between patients with GD and control subgroups, based on sexual orientation and stratified by birth-assigned sex, were examined with Kruskal–Wallis test, as it has been previously observed that homosexual controls (based on birth-assigned sex) demonstrate higher symptoms of gender dysphoria on GD evaluation scales.

To identify an appropriate TCS threshold value for clinical use, Receiver Operating Characteristic (ROC) curves were constructed and sensitivity and specificity values were calculated for different TCS thresholds, separately for each birth-assigned sex. Correlations between TCS and its factors with UGDS and GIDYQ-AA were examined with Spearman’s ρ.

Statistical significance was set at *p* < 0.05. Internal consistency, confirmatory factor analysis and multiple-group confirmatory factor analysis were calculated with R version 3.5.1. The Statistical Package for the Social Sciences (IBM SPSS Statistics) version 25 was used for all other analyses.

## Results

Sociodemographic data is presented in Table 1. The rate of birth-assigned sex did not differ between patients with GD and controls (39.3% vs. 31.4% for aM; 60.7% vs. 68.6% for aF, χ^2^ derived p = 0.089). Due to the recruitment approach of the control group through universities, education level in this group was significantly higher, compared to patients with GD (93 vs. 30.4% with higher education; χ^2^ derived p < 0.001). Furthermore, groups differed significantly in age and occupation status and, as expected, in satisfaction with body appearance (median score 15 vs. 70; GD vs. controls, Mann–Whitney derived p < 0.001, r (effect size) = − 0.66) and gender incongruence (median score 91 vs. 4; GD vs. controls; Mann–Whitney derived p < 0.001, r = − 0.71).

**Table 1:** Sociodemographic data of study population.

	Controls	Patients with GD	p^1^
	N (%)	N (%)	
**Birth-assigned sex**			
Male	139 (31.4)	53 (39.3)	0.089
Female	304 (68.6)	82 (60.7)	

	*N* = *443*	*N* = *135*	
**Age**			
< 18	1 (0.2)	14 (10.4)	< 0.001
18–24	111 (25.1)	63 (46.7)	
25–29	142 (32.1)	25 (18.5)	
30–39	139 (31.4)	20 (14.8)	
40–49	38 (8.6)	9 (6.7)	
50–59	11 (2.5)	2 (1.5)	
60–69	1 (0.2)	2 (1.5)	
**Education**			
Primary/high school	31 (7)	94 (69.6)	< 0.001
University/college	411 (93)	41 (30.4)	
**Occupation**			
Employed	249 (56.6)	52 (40.9)	< 0.001
Sick leave	14 (3.2)	15 (11.8)	
Unemployed	23 (5.2)	28 (22)	
Parental leave	8 (1.8)	0 (0)	
Other	146 (33.2)	32 (25.2)	
Median (IQR^4^)			
Satisfaction with body appearance^2^	70 (27)	15 (27)	< 0.001
Gender incongruence^3^	4 (14)	91 (23)	< 0.001

^1^Chi-square or Mann–Whitney.

^2^“How satisfied are you with your body?” (Score 1 – 100, 1 = very unsatisfied).

^3^“To what extend is your body’s appearance in line with your inner identity?” (Score 1–100, 1 = completely in line).

^4^Interquartile range.

The internal consistency of the TCS scale was analyzed with McDonald’s ω, which was 0.962 for the total scale, 0.985 for the *appearance congruence* factor and 0.784 for the *gender identity acceptance* factor. Regarding the total study population, confirmatory factor analysis supported the two-factor model of the TCS scale, where all questions loaded strongly on their factors. CFI was 0.974, SRMR 0.022 and RMSEA 0.049, indicating a good fit between the model and the observed data. Standardized factor loadings are provided in Table 2.

**Table 2:** Confirmatory factor analysis (CFA) derived factor loadings.

TCS Question (number)	Factor 1*	Factor 2*
My physical body represents my gender identity (5)	0.967	
I experience a sense of unity between my gender identity and my body (2)	0.961	
The way my body currently looks does not represent my gender identity (6)	0.951	
My physical appearance adequately expresses my gender identity (3)	0.943	
I do not feel that my appearance reflects my gender identity (8)	0.935	
I am generally comfortable with how others perceive my gender identity when they look at me (4)	0.931	
I am happy with the way my appearance expresses my gender identity (7)	0.929	
My outward appearance represents my gender identity (1)	0.910	
I feel that my mind and body are consistent with one another (9)	0.909	
I am happy that I have the gender identity that I do (11)		0.887
I have accepted my gender identity (12)		0.726
I am not proud of my gender identity (10)		0.608

Correlation between Factor 1 and Factor 2: 0.473.

*Standardized coefficients.

Regarding MGCFA for birth-assigned sex, the baseline model, which freely estimated factor loadings and intercepts across birth-assigned sex, had adequate fit (χ^2^ [df = 106] = 565.85, CFI = 0.954 and RMSEA = 0.093), indicating configural invariance (similar factor structure for aM and aF). Next, we fixed the factor loadings to equality across aM and aF (χ^2^ [116] = 601.34, CFI = 0.950 and RMSEA = 0.092), which indicated metric invariance (similar factor loadings for aM and aF) since CFI changed < 0.01 and RMSEA changed < 0.015 compared to the baseline model. Finally, we fixed the intercepts to equality across aM and aF and this model (χ^2^ [126] = 627.33, CFI = 0.949 and RMSEA = 0.088) indicated scalar invariance (similar item intercepts for aM and aF) since CFI changed < 0.01 and RMSEA changed < 0.015 compared to the previous model. When running MGCFA for patients with GD and controls in the same way, the baseline model had adequate fit (χ^2^ [106] = 400.64, CFI = 0.925 and RMSEA = 0.099), indicating configural invariance. Thereafter, the factor loadings were fixed to equality across aM and aF (χ^2^ [116] = 495.21, CFI = 0.904 and RMSEA = 0.107). Since CFI changed > 0.01 compared to the baseline model the criterion of metric invariance was not fulfilled.

**Figure 1:**
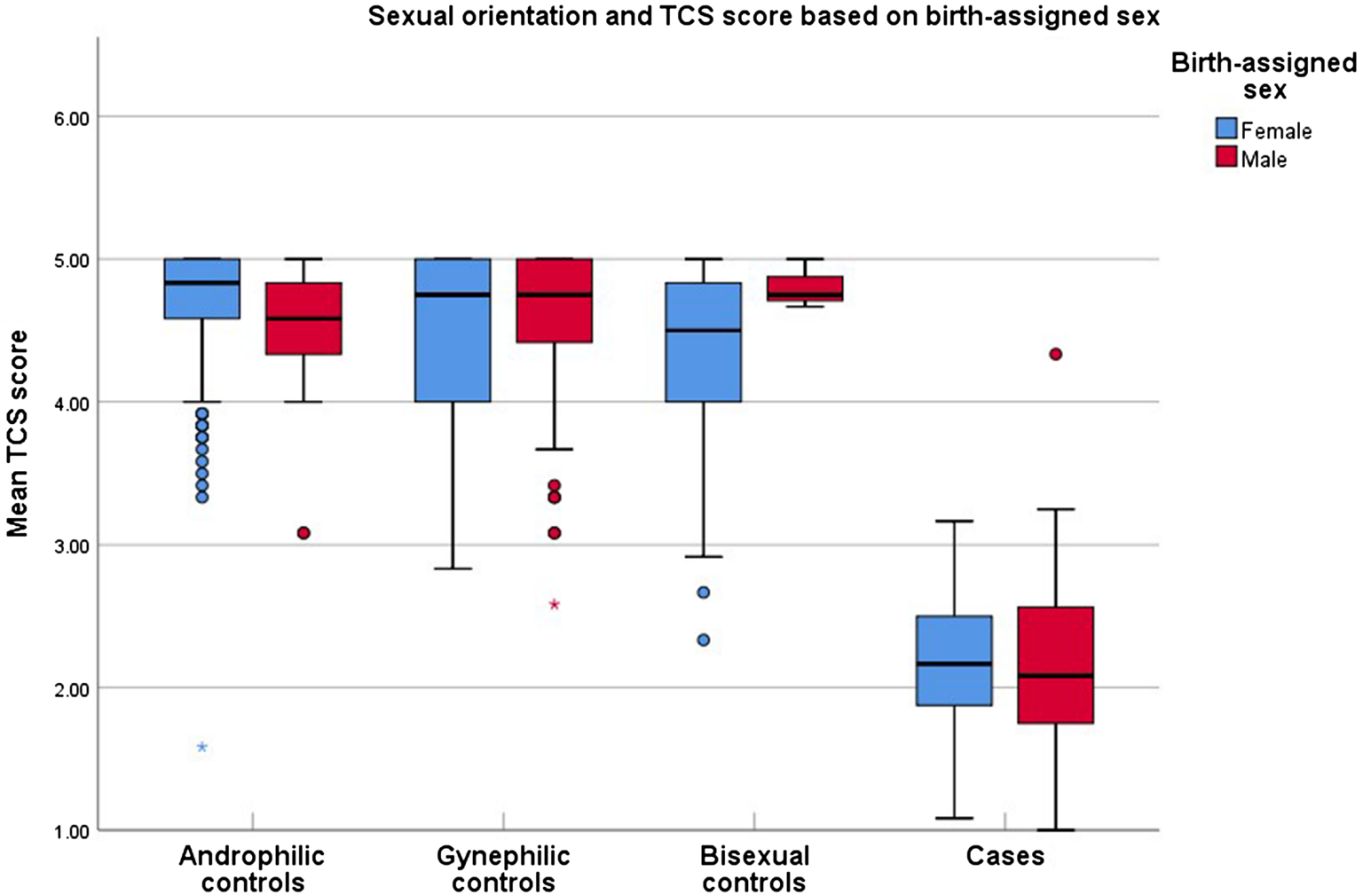
TCS scores between case and control subgroups with different sexual orientation, stratified by birth-assigned sex. *Only androphilic, gynephilic and bisexual patients with GD included.

Table 3 illustrates differences in GD evaluation scales between study groups. Patients with GD, stratified by birth-assigned sex, had significantly lower TCS scores compared to controls (aM median scores patients with GD vs. controls: 2.00 vs. 4.75, Mann–Whitney derived p < 0.001, r = − 0.76; aF median score GD vs. controls: 2.17 vs. 4.75, Mann–Whitney derived p < 0.001, r = − 0.7). The same applied for the TCS factors *appearance congruence* (aM median scores GD vs. controls: 1.56 vs. 4.89, Mann–Whitney derived p < 0.001, r = − 0.79; aF median score GD vs. controls: 1.67 vs. 4.89, Mann–Whitney derived p < 0.001, r = − 0.72) and *gender identity acceptance* (aM median scores GD vs. controls: 3.67 vs. 4.33, Mann–Whitney derived p = 0.003, r = − 0.22; aF median score GD vs. controls: 4 vs. 4.67, Mann–Whitney derived p < 0.001, r = − 0.28) as well as when comparing each TCS question separately. Patients with GD had significantly lower scores in all 12 TCS items compared to controls, except for question 10 in aM (median scores GD vs. controls: 3 vs. 4, Mann–Whitney derived p = 0.215). Similar results were observed regarding the UGDS (aM median scores GD vs. controls: 50.5 vs. 13, Mann–Whitney derived p < 0.001, r = − 0.64; aF median score GD vs. controls: 56 vs. 18, Mann–Whitney derived p < 0.001, r = − 0.54) and the GIDYQ-AA (aM median scores GD vs. controls: 2.52 vs. 4.7, Mann–Whitney derived p < 0.001, r = − 0.63; aF median score GD vs. controls: 2.19 vs. 4.7, Mann–Whitney derived p < 0.001, r = − 0.58). Results of the analyses named above remained unaltered after stratifying for sexual orientation (Table 4 and Fig. 1). Androphilic, gynephilic and bisexual patients with GD had TCS scores more indicative of GD, compared to corresponding controls (aM median scores GD vs. androphilic vs. gynephilic vs. bisexual controls: 2.08 vs. 4.58 vs. 4.75 vs. 4.75, Kruskal–Wallis derived p < 0.001; aF median scores GD vs. androphilic vs. gynephilic vs. bisexual controls: 2.17 vs. 4.83 vs. 4.75 vs. 4.5, Kruskal–Wallis derived p < 0.001). Additionally, 7% of aF and 2.1% of aM controls identified themselves as asexual, uncertain or “other” while among patients with GD the equivalent rate was 33% for aF and 7.7% for aM.

**Table 3:** Median and interquartile range (IQR) values for the Transgender Congruence Scale (TCS), as total scale as well as per factor and per item, the Gender Identity/Gender Dysphoria Questionnaire for Adolescents and Adults (GIDYQ-AA) and the Utrecht Gender Dysphoria Scale (UGDS) among controls and patients with GD, stratified by birth-assigned sex.

TCS	Birth-assigned female			Birth-assigned male		
	Controls Median (IQR**)	Patients with GD Median (IQR)	p-value*	Controls Median (IQR)	Patients with GD Median (IQR)	p-value*
	*N* = *304*	*N* = *82*		*N* = *139*	*N* = *53*	
Total scale	4.75 (0.58)	2.17 (0.67)	< 0.001	4.75 (0.58)	2.00 (0.77)	< 0.001
Appearance congruence	4.89 (0.44)	1.67 (0.81)	< 0.001	4.89 (0.56)	1.56 (0.89)	< 0.001
Gender identity acceptance	4.67 (1)	4 (1.33)	< 0.001	4.33 (1.33)	3.67 (1.83)	0.003
**Questions (number)**						
1	5 (1)	2 (2)	< 0.001	5 (0)	2 (1)	< 0.001
2	5 (0)	1 (0)	< 0.001	5 (0)	1 (1)	< 0.001
3	5 (1)	2 (1)	< 0.001	5 (1)	2 (1)	< 0.001
4	5 (0)	2 (1)	< 0.001	5 (0)	1 (1)	< 0.001
5	5 (0)	1 (0)	< 0.001	5 (0)	1 (1)	< 0.001
6	5 (0)	1 (1)	< 0.001	5 (0)	1 (1)	< 0.001
7	5 (1)	2 (1)	< 0.001	5 (1)	1 (1)	< 0.001
8	5 (0)	2 (1)	< 0.001	5 (0)	1 (1)	< 0.001
9	5 (1)	1 (1)	< 0.001	5 (1)	1 (1)	< 0.001
10	5 (2)	4 (2)	< 0.001	4 (2)	3 (2)	0.215
11	5 (1)	4 (1)	< 0.001	4 (2)	3.5 (3)	0.004
12	5 (0)	5 (1)	< 0.001	5 (0)	5 (1)	< 0.001

GIDYQ-AA	*N* = *291*	*N* = *43*		*N* = *136*	*N* = *25*	
Total scale	4.7 (0.3)	2.19 (0.44)	< 0.001	4.7 (0.29)	2.52 (0.63)	< 0.001

UGDS	*N* = *280*	*N* = *36*		*N* = *135*	*N* = *24*	
Total scale	18 (9)	56 (9.75)	< 0.001	13 (3)	50.5 (14.5)	< 0.001

*Mann–Whitney. **Interquartile range.

**Table 4:** Sexual orientation and TCS scores among patients with GD and control subgroups, stratified by sexual orientation.

Birth-assigned sex	Androphilic^1^ controls	Gynephilic^2^ controls	Bisexual^3^ controls	Patients with GD^1,2,3^	p*
**Female**					
N (%)	209 (68.9)	24 (7.9)	49(16.2)	55 (67.2)	
TCS Median (IQR^4^)	4.83 (0.42)	4.75 (1)	4.5 (0.92)	2.17 (0.75)	< 0.001^a^
**Male**					
N (%)	19 (13.7)	114 (82.1)	3 (2.2)	48 (92.3)	
TCS Median (IQR^4^)	4.58 (0.5)	4.75 (0.6)	4.75 (-)	2.08 (0.82)	< 0.001^b^

*Kruskal–Wallis.

^1^Attracted only/mostly to men.

^2^Attracted only/mostly to women.

^3^Attracted to men and women equally.

^4^Interquartile range.

^a^Differences in TCS median scores among control subgroups were only significant between androphilic and bisexual controls (p < 0.05, Mann–Whitney).

^b^Differences in TCS median scores among control subgroups were not significant (Mann–Whitney).

**Figure 2:**
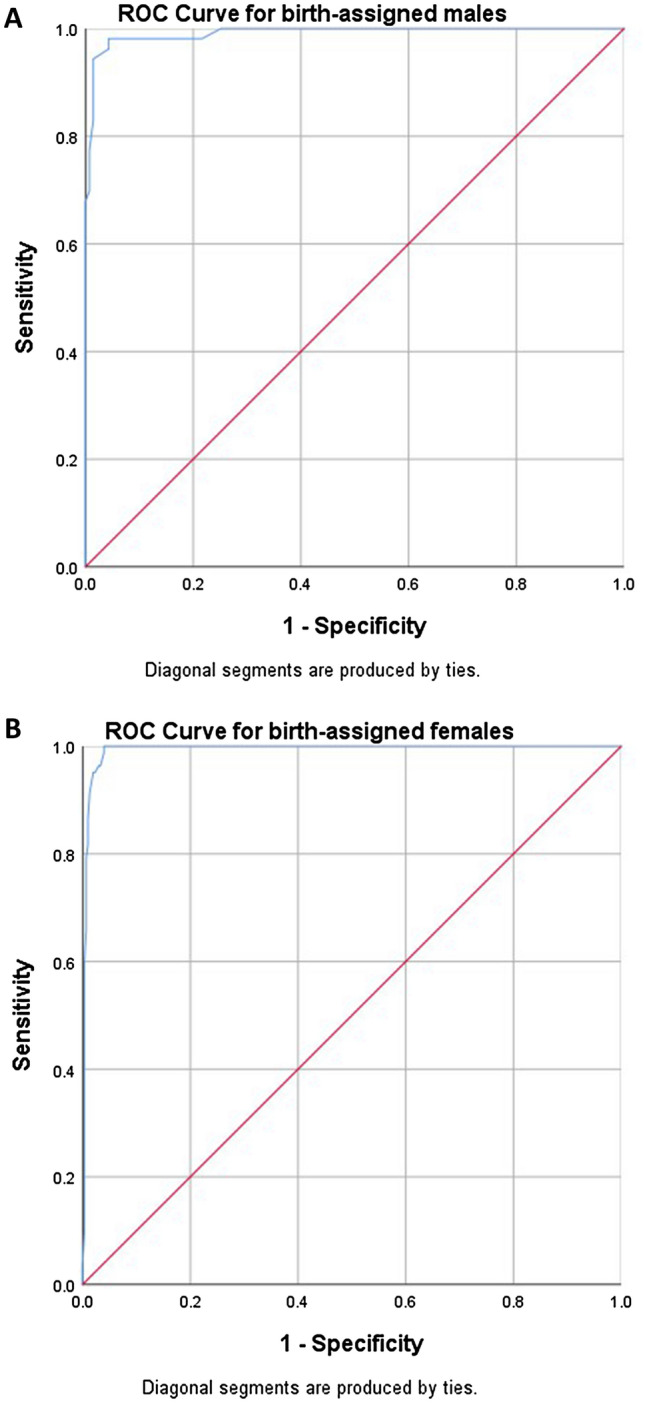
(**a**,**b**) Receiver Operation Characteristic (ROC) curves for aM and aF.

Due to previous reports on differences in GD evaluation scores depending on the birth-assigned sex, different ROC curves were constructed for aM and aF (Fig. 2a,b). Patients with GD were assumed true positive and controls true negative. Area under the Curve (AUC) for aM was 0.991 (95% CI 0.981–1.00) and for aF 0.994 (95% CI 0.987–1.000). Table 5 presents calculated sensitivity and specificity for different TCS cut-off values, separately for birth-assigned sexes. A TCS mean value of 3 would provide a sensitivity of 94.3% and specificity of 98.6% for aM, while the same TCS cut-off for aF would provide a sensitivity of 95.1% and specificity of 98%.

**Table 5:** Sensitivity and specificity for different TCS mean values in birth-assigned males and females.

TCS mean value cut-offs	Sensitivity (%)	Specificity (%)
**Birth-assigned males**		
2.33	67.9	100.0
2.56	77.4	99.3
3.00	94.3	98.6
3.29	98.1	95.7
4.35	100	74.8
**Birth-assigned females**		
1.54	3.7	100.0
2.29	59.8	99.7
2.63	79.3	99.3
2.79	86.6	99.0
2.88	91.5	98.7
3.00	95.1	98.0
3.04	95.1	97.7
3.09	96.3	97.0
3.29	100	96.1

Finally, the TCS was significantly correlated to UGDS (Spearman’s ρ; male version − 0.751, female version − 0.730, p < 0.001) and GIDYQ-AA (Spearman’s ρ; male version 0.792, female version 0.770, p < 0.001) (Table 6).

**Table 6:** Spearman’s correlation coefficients (ρ) between TCS and UGDS as well as GIDYQ-AA.

	Male version			Female version		
	N	ρ	p	N	ρ	p
**UGDS**						
TCS	159	− 0.751	< 0.001	316	− 0.730	< 0.001
Appearance congruence		− 0.754	< 0.001		− 0.697	< 0.001
Gender identity acceptance		− 0.395	< 0.001		− 0.505	< 0.001
**GIDYQ-AA**						
TCS	161	0.792	< 0.001	334	0.770	< 0.001
Appearance congruence		0.809	< 0.001		0.756	< 0.001
Gender identity acceptance		0.439	< 0.001		0.508	< 0.001

## Discussion

Results of the present study provide evidence that the Swedish version of TCS is a non-binary scale with good psychometric properties and can distinguish individuals with gender dysphoria. The scale had sound discriminatory qualities as a whole and even in its separate factors of *appearance congruence* and *gender identity acceptance*, in both birth-assigned sexes. TCS had also high concurrent validity when compared to the UGDS and GIDYQ-AA.

It has previously been reported that non-heterosexual men and women, defined on the basis of birth-assigned sex, may exhibit significantly more gender dysphoria than heterosexual individuals^6^. In our study, the only significant difference in terms of gender dysphoria symptoms, among sexual orientation control subgroups, was observed between androphilic and bisexual female controls, where bisexuals scored lower on TCS. However, the difference in median TCS scores between these two groups was only 0.33 points, while the overall TCS median score among controls was at least twice as high as for patients with GD. Therefore, the present data do not support the need of considering sexual orientation when evaluating gender dysphoria with the TCS scale.

Confirmatory factor analysis supported the two-factor model, in accordance to the original TCS study^7^. Multiple-group confirmatory factor analysis suggested measurement invariance between birth-assigned sexes. Only configural invariance was shown between patients with GD and controls. When checking for metric invariance between patients with GD and controls, the decrease in CFI can possibly be explained by the fact that the TCS was constructed to detect GD and is thus more fitted for individuals with symptoms of gender dysphoria.

Sensitivity and specificity cut-off values were similar between birth-assigned sexes, since a TCS mean value between 3 and 3.29 yielded a sensitivity of 94.3–98.1% and a specificity of 98.6–95.7% for aM, while for aF the corresponding values were 95.1–100% for sensitivity and 98–96.1% for specificity, for the same TCS cut-offs. These results further support the notion of TCS as a non-binary and sex-independent scale for assessing gender dysphoria, even if we did not explicitly study the validity of the scale in non-binary persons.

This study provides supportive evidence for using the scale in clinical practice in the Swedish gender dysphoria evaluation centers. A large proportion of transgender people seeking care in these clinics are interested in gender affirming hormonal therapy and some sort of surgery (Axfors C et al., submitted for publication). Before the development of TCS we lacked an instrument that could assess treatment results in persons that had undergone some but not all gender affirming treatments, since the UGDS and the GIDYQ are problematic in these cases. TCS has increasingly been used by clinicians who assess patient with GD in Sweden and is also included as a Patient Reported Outcome Measure (PROM) in the national quality register for gender dysphoria (https://konsdysforiregistret.se/). Moreover, it has been shown that patients perceive less distress, greater satisfaction with their body and better mental health and life satisfaction after gender affirming surgery^15^. Therefore, instruments that assess satisfaction, quality of life and body perception are needed, in order to evaluate treatment results and properly follow-up transgender individuals, as described in a systematic review by Barone et al.^16^. In fact, the research group that constructed the TCS suggests its use in various time-points during the course of treatment, as a measure of within-individual congruence changes^7^. Further studies should investigate the scale’s function in terms of following up treatment effect and patient satisfaction.

More recently, a new scale has emerged, the Gender Congruence and Life Satisfaction Scale (GCLS) which includes measurement of gender congruence, mental health and quality of life and has similar advantages with the TCS, but the Swedish version of the scale has not yet been validated^17^.

### Strengths and limitations

This is the first study to validate the Swedish version of the TCS in transgender individuals seeking healthcare. Although the sample size on which the development of the TCS tool was based on was larger, the present study still had a reasonable number of participants with probable gender dysphoria recruited from various GD evaluation centers as well as a large control group. The high recruitment rate and the relatively large amount of available background data of study participants should be considered as study strengths.

Study groups were quite homogeneous by implementing exclusion of transgender people on hormonal treatment as well as controls that reported gender affirming treatment or the wish for it. This should have counterbalanced the over-representation of gender dysphoric controls that were willing to enroll to the study, since it seems plausible that these individuals may have been more interested in participating in the study. However, this aspect should still be considered as a study limitation. Nevertheless, a possible misclassification towards this direction should only have debilitated the observed significant differences between study groups. Study results should be interpreted with caution, in terms of study generalizability, considering the rather young study sample in both groups. Indeed, the vast majority of participants were younger than 40, while other studies in Sweden report older study samples of individuals applying for gender affirming medical treatment^18^. On the other hand, the study sample assessed upon construction of the TCS scale was also older^7^, implying that the scale fits groups of various age ranges. Furthermore, it has been reported that the number of young participants seeking care in gender dysphoria centers has gradually increased over time^19^.

The significantly higher proportion of controls with higher education depends mainly on the recruitment strategy as well as study setting, since Uppsala inhabitants generally have a higher educational level, compared to the Swedish population in general.

Another study limitation is the lack of gender dysphoria diagnosis upon enrollment of patients with GD. According to the DSM-5 definition, gender dysphoria criteria should last for at least six months. However, almost all patients initiating a gender dysphoria evaluation in one of the centers can be considered having major gender dysphoria symptoms even if they do not fulfill the diagnostic criteria. Moreover, the average waiting time for initiation of evaluation in a gender dysphoria center in Sweden is approximately one year, which suggests that these symptoms are persistent over time. Finally, in this study we did not have data on self-reported non-binary identification, which would allow us to specifically evaluate the function of the scale in this population.

## Conclusion

In this study, the Swedish version of the Transgender Congruence Scale has been validated in transgender individuals seeking healthcare in gender dysphoria evaluation centers. TCS had good psychometric properties and could distinguish individuals with gender dysphoria, in both birth-assigned sexes. A TCS mean score with a cutoff of 3.0 is suggested for screening of GD among persons with gender dysphoria symptoms. More research is warranted for the utility of the scale during the follow-up of patients receiving gender affirming treatment, as the sex-independent structure of the scale is well suited for this purpose.

## Data Availability

The data that support the findings of this study are available upon reasonable request from the corresponding author. The data are not publicly available due to privacy and ethical restrictions. The data were taken from our own studies.
